# The Role of Prenatal Ultrasound and Added Value of Post‐Mortem Radiographic Imaging With X‐Ray and CT in Suspected Fetal Skeletal Dysplasia

**DOI:** 10.1002/pd.6732

**Published:** 2024-12-15

**Authors:** Katri Rajala, Sanna Toiviainen‐Salo, Outi Mäkitie, Vedran Stefanovic, Laura Tanner

**Affiliations:** ^1^ Department of Clinical Genetics Kuopio University Hospital Kuopio Finland; ^2^ Department of Pediatric Radiology HUS Medical Imaging Center, Radiology Helsinki University Hospital and University of Helsinki Helsinki Finland; ^3^ Faculty of Medicine Research Program for Clinical and Molecular Metabolism University of Helsinki Helsinki Finland; ^4^ Children's Hospital and Pediatric Research Center University of Helsinki and Helsinki University Hospital Helsinki Finland; ^5^ Folkhälsan Research Center Helsinki Finland; ^6^ Department of Obstetrics and Gynecology Fetomaternal Medical Center Helsinki University Hospital and University of Helsinki Helsinki Finland; ^7^ HUSLAB Department of Clinical Genetics Helsinki University Hospital Helsinki Finland; ^8^ Department of Medical and Clinical Genetics University of Helsinki Helsinki Finland

## Abstract

**Objective:**

This study aims to assess the diagnostic value of post‐mortem radiographic imaging compared with prenatal ultrasound in suspected fetal skeletal dysplasias in a large Finnish cohort.

**Method:**

Prenatal ultrasound findings and their association with post‐mortem radiographic imaging were evaluated in a cohort of 36 fetuses with prenatally suspected skeletal dysplasia.

**Results:**

Prenatal ultrasound performed well in detecting skeletal dysplasias and severe forms of the disease. Additional radiographic imaging was performed post‐mortem in 16/27 terminated pregnancies. Post‐mortem X‐ray and 3D‐CT detected several features not seen with US. They were superior to US in identifying spinal and thoracic anomalies and performed better in discovering fractures and deformities of long bones. In addition, disease‐specific findings became more accurate with X‐ray/CT, especially in the group of true skeletal dysplasias (14/18, 77.8%). Post‐mortem X‐ray and CT increased phenotypic data and facilitated interpretation of genetic findings.

**Conclusion:**

Post‐mortem X‐ray and CT offer additional information supporting the diagnostic process. Detailed phenotypic data are important in interpreting the results of genetic analyses and in assessing the recurrence risk in future pregnancies. Complementary imaging methods including post‐mortem radiography are therefore recommended.

**Summary**

- What's already known about this topic?
  ◦ Prenatal ultrasound performs well in detecting skeletal dysplasias.
  ◦ Prenatal CT has been shown to be better in evaluating fetal skeletal abnormalities, but fetal radiation exposure limits its feasibility in ongoing pregnancy.
- What does this study add?
  ◦ X‐ray and CT offer additional information supporting the diagnostic process after termination of pregnancy or fetal demise.
  ◦ Detailed phenotypic data are important for the interpretation of results of molecular genetic testing and assessment of recurrence risk in future pregnancies.

## 1 Introduction

Skeletal dysplasias are a group of disorders affecting the development of bone and cartilage. Over 770 different disorders have been recognized to date with a total birth incidence of 1/5000. The genetic spectrum of skeletal dysplasias can differ significantly between populations. The most severe forms are associated with significant perinatal morbidity and mortality [1, 2, 3, 4].

The primary diagnostic tool to identify fetal skeletal disease is prenatal ultrasound (US). Usually the first abnormal finding observed with US is shortening of the long bones. Other signs indicating skeletal disorder are bowing, poor mineralization, or fractures of the bones along with abnormalities in flat bones, vertebrae, and thorax [5]. Nuchal translucency (NT) or fetal hydrops can also be associated with skeletal dysplasia [6].

Assessing the degree of severity of potential fetal skeletal dysplasia during pregnancy is often challenging due to the large number of possible diagnoses with variable and overlapping phenotypes. In the absence of an exact genetic diagnosis, the decision‐making relies on clinical assessment with US [2]. Prenatal US has only 40%–68% diagnostic accuracy in detecting skeletal dysplasia [5, 7, 8, 9] and poor visibility due to for example maternal obesity can complicate the process [10]. With appropriate criteria, US is quite accurate in distinguishing between lethal and non‐lethal types of the disease. The most accurate predictors of lethal skeletal dysplasia are 3D fetal lung volumes (< 5th percentile for gestational age), ratios of femur length to abdominal circumference (FL/AC < 0.16) and chest circumference to abdominal circumference (TC/AC < 0.6). Other features potentially indicating lethal disease include a bell‐shaped thorax, short ribs, severe femoral shortening, polyhydramnion and multiple fractures or underossification of the spine [11, 12, 13].

When fetal skeletal disease is suspected, obtaining an accurate diagnosis is crucial for families' decision‐making concerning for example continuation of the pregnancy or abstaining from intensive postnatal care in case of poor prognosis. The diagnostic process is heavily influenced by local protocols concerning termination of pregnancy, which may be very variable. Molecular genetic testing can considerably expand diagnostic options [14, 15]. However, genetic analyses are time‐consuming, and suspicion of skeletal disease may also rise rather late in pregnancy. Therefore, a specific diagnosis may not be available within the legal limit for the termination of the pregnancy. Obtaining the exact diagnosis after termination is useful for the families and it enables counseling and invasive diagnostic testing in further pregnancies if needed.

In addition to US, various imaging methods have been used. Fetal magnetic resonance imaging (MRI) has been useful during pregnancy in assessing fetal lung volumes to predict lethality and to evaluate brain, spine, and cartilage defects [16, 17]. Low‐dose CT and 3D images could also provide more information on the fetal skeleton when molecular genetic testing is not available, or the findings are difficult to interpret [18]. Radiographic imaging and histological findings have been helpful in determining the type of skeletal disease after fetal demise or termination of the pregnancy [19].

The aim of this study was to assess the diagnostic value of prenatal ultrasound and post‐mortem radiographic imaging with X‐ray and CT in suspected fetal skeletal dysplasias in a large Finnish cohort.

## 2 Material and Methods

This study was based on a retrospectively collected cohort, involving pregnancies with prenatally suspected or diagnosed fetal skeletal dysplasias between January 2013 and March 2020 at the Fetomaternal Medical Center (FMC) at Helsinki University Hospital. FMC is a tertiary institution for fetal medicine and its catchment area covers almost one third of the Finnish population. Clinical details about pregnancies, including US and genetic findings, have been presented in our previous publication [4]. In the present study, we carried out a more extensive evaluation of the US findings and their association with radiographic imaging findings in this same cohort. This research was approved by Helsinki University Central Hospital (no. 4199, November 4, 2020). As this was a retrospective study utilizing hospital register data, informed consent or separate ethical approval was not required.

In Finland, all pregnant women are offered prenatal ultrasound screening free of charge. First trimester screening is offered between 10 + 1 and 13 + 6 gestational weeks and second trimester morphology screening between 18 + 0 and 21 + 6 weeks. If fetal abnormality is suspected, families are referred to a fetal diagnostic unit for further evaluation and counseling. Invasive diagnostic testing (either chorionic villus sampling or amniocentesis) is offered if genetic etiology is suspected. According to the Finnish law, termination of pregnancy (TOP) is allowed before 24 weeks of pregnancy if a severe fetal abnormality (structural anomaly, genetic abnormality or other severe fetal disease) is diagnosed with a reliable method.

Ultrasound in our cohort was performed by an experienced perinatologist at the fetal diagnostic unit and both two‐dimensional (2D) and three‐dimensional (3D) imaging were used. Fetal MRI was available but it has not been routinely used in cases with suspected skeletal dysplasia and was not applied in this cohort.

After termination of pregnancy or after fetal demise, radiographic imaging using ionizing radiation (X‐ray and/or CT with 3D reformats) was performed. In cases where the diagnosis had not been genetically confirmed during the pregnancy, fetal autopsy was performed and DNA samples were obtained for genetic analyses. Liveborn children were evaluated by a pediatrician and skeletal findings were evaluated by X‐ray. All imaging was performed in normal clinical settings. X‐ray and CT were reviewed concurrently, and the findings were not separated in the report.

We classified the diagnoses as skeletal dysplasias if they were included in the recent version of Nosology (2023; [3]), while diagnoses resembling skeletal disease but absent from the Nosology were considered as a separate group that should be considered in differential diagnostics.

## 3 Results

### 3.1 Cohort Characteristics

**FIGURE 1:**
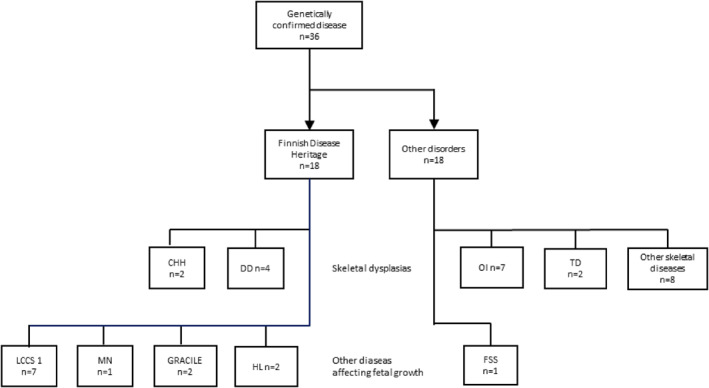
Genetically confirmed diseases of our cohort. CHH, Cartilage‐hair hypoplasia; DD, Diastrophic dysplasia; FSS, Freeman‐Sheldon syndrome; HL, Hydrolethalus; LCCS 1, Lethal congenital contracture syndrome 1; MN, Mulibrey nanism; OI, Osteogenesis imperfecta; TD, Thanatophoric dysplasia. Figure is modified from our previous article (Rajala et al. 2022).

From a total cohort of 121 pregnancies with suspected fetal skeletal dysplasia, genetic confirmation was obtained in 34 cases. In two additional cases, fetal phenotypes were consistent with the diagnosis of skeletal dysplasia, but genetic findings were classified as variants of unknown significance and further analyses are pending. Of these 36 cases, 23 (63.9%) were true skeletal dysplasias (Table 1a), whereas 13 were diagnosed with other diseases affecting fetal growth and often mimicking skeletal disease (Table 1b). Half of the diagnoses in the 36 cases belonged to the Finnish Disease Heritage (FDH), a group of rare inherited disorders enriched in Finland. The distribution of diagnoses is presented in Figure 1. Nine cases led to live birth and 27/36 (75%) pregnancies were terminated.

**TABLE 1a:** Radiological imaging findings of the 23 cases of true skeletal dysplasias included in the study. Added values of X‐ray and CT findings are bolded.

	Diagnosis and causative gene	GA	Ultrasound	X‐ray/CT	Method with disease specific findings
1	Diastrophic dysplasia (SLC26A2)	H12 + 2	Long bones short. Extremities flexed, feet extended and 'hitchhiker's thumbs'.	X‐ray and CT: Long bones short. **Tibiae, ulnae and radii bowed. Clubfoot in left** **.**	US supported by X‐ray and CT
2	Diastrophic dysplasia (SLC26A2)	H13 + 3	Long bones short, malposition of feet, 'hitchhiker's thumbs'.	X‐ray and CT: **Long bones** short, **wider and femora bowed. Ribs short**, not horizontal.	US supported by X‐ray and CT
3	Diastrophic dysplasia (SLC26A2)	H21 + 2	Long bones short (5–6 weeks smaller than GA). Clubfeet, sandal gap and 'hitchhiker's thumbs'. Small chin.	X‐ray and CT: Long bones short, **femora slightly bowed**, thumbs and first toes in abnormal position. Clubfeet.	US supported by X‐ray and CT
4	Diastrophic dysplasia (SLC26A2)	H20 + 6	Long bones short (5 weeks smaller than GA), right femur and left fibula bowed.	X‐ray and CT: **Long bones** short and **wide. Humeri** and shins **bowed**. Both wrists flexed, **'hitchhiker's thumbs'**.	X‐ray and CT
5	Osteogenesis imperfecta, severe perinatal form (Sillence type 2), COL1A1‐related	H20 + 6	Long bones short, femora 6 weeks smaller than GA. Feet normal‐sized. Thorax bell‐shaped, poor ossification of the skull. AC and HC normal size.	X‐ray and CT: All **long bones** short, **wide, bowed and deformed. Deformed and bowed ribs, suspected fractures**. Skull bones not visible.	X‐ray and CT
6	Osteogenesis imperfecta, non‐deforming (Sillence type 1), COL1A1‐related		No US findings during pregnancy.	No radiographic imaging.	(Familial disease)
7	Osteogenesis imperfecta, severe perinatal form (Sillence type 2), COL1A1‐related	H21 + 2	Long bones short, left femur, tibia and bones in upper extremities bowed. Rib fractures, abnormally shaped chest. Clubfoot on right.	Postnatal x‐ray: Humeri bowed, wide and deformed. Forearm bones bowed. **Abnormally shaped scapula and acromion**. Femora, tibiae and fibulae wide and with abnormal bone structure. **Multiple healing fractures**. Ribs deformed because of multiple fractures.	X‐ray and CT (some signs in US)
8	Osteogenesis imperfecta, progressively deforming (Sillence type 3), COL1A1‐related	H21 + 5	Long bones mildly short (2 weeks shorter than GA). Normally shaped bones.	Postnatal x‐ray: **Old fracture in right femur** (may not have been visible in US).	X‐ray and CT
9	Osteogenesis imperfecta severe perinatal form (Sillence type 2), COL1A1‐related^a^	H20 + 3	Long bones short. Femora 6 weeks smaller than GA. Humeri, fibulae and radii 4–5 weeks smaller. Short ribs. Abnormal position of ankles.	X‐ray and CT: **Multiple deformities and fractures in ribs. Humeri and radii bowed by fractures. Scarce ossification of skull.**	X‐ray and CT
10	Osteogenesis imperfecta severe perinatal form (Sillence type 2), COL1A2‐related^a^	H20 + 1	Femora, humeri, tibiae and fibulae short. Bones in lower extremities bowed.	X‐ray and CT: **Multiple fractures in ribs. Femora** short **with fractures**. Tibiae bowed.	X‐ray and CT
11	Osteogenesis imperfecta severe perinatal form (Sillence type 2), COL1A2‐related^a^	H20 + 4	Long bones short, femora 6 weeks smaller than GA. Femora and humeri bowed. Ribs short. Head angular and poorly mineralized.	X‐ray and CT: Long bones short and bowed. **Fractures seen in lower and upper extremities. Fractures in ribs, right clavicle and scapula. Suspected fractures in vertebra.** Only basal structures of the skull visible.	X‐ray and CT (some signs in US)
12	Thanatophoric dysplasia (FGFR3)	H20 + 2	Long bones short. Radii and fibulae shortest (5–6 weeks smaller than GA). Slightly small chest.	No radiographic imaging.	Genetic testing (supported by US)
13	Thanatophoric dysplasia (FGFR3)	H13 + 6	Femora and humeri short and bowed, small chest.	No radiographic imaging.	Genetic testing (supported by US)
14	Spondyloepiphyseal dysplasia (COL2A1)	H22 + 0	Long bones short (2–3 weeks shorter than GA)	Postnatal x‐ray: **Long bones** short and **abnormally shaped, metaphyses wider. Short ribs. Abnormal vertebrae.**	X‐ray
15	Spondyloepiphyseal dysplasia (COL2A1)	H31 + 5	US normal at second trimester screening. H31 + 5: Short long bones, femora 3 weeks smaller than GA. Polyhydramnion.	Postnatal x‐ray: **Abnormal cervical vertebrae. Diaphyses of humeri, radii and ulnae** are short and **wide**.	X‐ray
16	Osteochondrodysplasia congenita (ACAN)	H20 + 3	Long bones 3 weeks smaller than GA.	X‐rays and CT: Short long bones.	Genetic testing
17	Osteochondrodysplasia congenita (COL2A1)	H13 + 6	Narrow thorax. Femora and humeri 2 weeks smaller than GA. Right femur shorter and bowed. Clubfoot on right.	X‐ray and CT: Ribs slightly short. Humerus and bones of forearm short. Right femur and tibiae short. **Less ossification nuclei in spine.**	X‐ray and CT
18	Short‐rib thoracic dysplasia 3 (DYNC2H1)	H20 + 0	Long bones short (2–3 weeks smaller than GA). Femora, tibiae and humeri bowed. Thorax slightly smaller than AC. Short ribs and narrow chest.	X‐ray and CT: Femora and humeri short, femora bowed. **Pelvic foils small.** Ribs short and horizontal.	X‐ray and CT
19	Type 1A Brachydactyly (IHH)	H20 + 5	Femora, humeri, tibiae and fibulae short. Thorax slightly smaller than AC.	Postnatal x‐ray: **Left arm x‐ray shows short middle phalanxes.**	X‐ray
20	Cartilage‐hair hypoplasia (RMRP)	H21 + 4	Twin pregnancy. Long bones short in both (3–4 weeks smaller than GA). Chest normal.	No radiographic imaging.	Genetic testing
21	Cartilage‐hair hypoplasia (RMRP)	H21 + 3	Long bones short (3 weeks smaller than GA).	No radiographic imaging.	Genetic testing
22	Spondylocostal dysostosis 5 (TBX6^b^)	H11 + 6	Edema widely under skin, NT 8.1 mm. 2 fingers extended. Poor visibility, obese mother.	X‐ray: **Small thorax, deformed ribs. Abnormal vertebrae.**	X‐ray and CT
23	Spondylocostal dysostosis 5 (TBX6^b^)	H11 + 2	NT 8.9 mm, edema widely under skin. Stiffness of lower extremities, other leg flexed. Short trunk, small chest.	X‐ray: **Abnormal vertebra.** Thorax abnormal, **ribs not visible.**	X‐ray and CT

^a^ Reclassified after new Nosology (2023).

^b^ Clinical significance is unknown, further analysis pending.

Abbreviations: AC, abdominal circumference; GA, gestational age; HC, head circumference; NT, nuchal translucence; TC, chest circumference.

**TABLE 1b:** Radiological imaging findings of the 13 cases of other diseases affecting fetal growth. Added value of X‐ray and CT findings are bolded.

	Diagnosis and causative gene	GA	Ultrasound	X‐ray/CT	Method with disease specific findings
24	Lethal congenital contracture syndrome 1 (GLE1)	H13 + 1	NT 3.2 mm. No fetal movements. Lower extremities straight, arms flexed, clubfeet.	No radiographic imaging.	Genetic testing (absent movements seen with US)
25	Lethal congenital contracture syndrome 1 (GLE1)	H14 + 2	Increased NT, pleural effusion and ascites. No fetal movements. Arms flexed, knees extended flexed hips. Unilaterally flexed wrist and ankle.	X‐ray: Long bones normal, large claviculas. Elbows and wrists in malposition. **Dislocated right hip.**	Genetic testing (absent movements seen with US)
26	Lethal congenital contracture syndrome 1 (GLE1)	H15 + 0	No fetal movements. Effusion widely under skin. Hips flexed and one knee extended. Contractures of arms.	No radiographic imaging.	Genetic testing (absent movements seen with US)
27	Lethal congenital contracture syndrome 1 (GLE1)	H15 + 0	No fetal movements. Fetal hydrops, pleural effusion. Femora and humeri slightly shortened. Hips flexed, knees straight, ankles extended. Arms flexed.	No radiographic imaging.	Genetic testing (absent movements seen with US)
28	Lethal congenital contracture syndrome 1 (GLE1)	H20 + 4	No fetal movements. Femora and humeri over 2 weeks smaller than GA. Angulated head. Fetal hydrops, pleural effusion. Elbows flexed, fingers in ulnar deviation. Knees straight, unilateral clubfoot.	No radiographic imaging.	Genetic testing (absent movements seen with US)
29	Lethal congenital contracture syndrome 1 (GLE1)	H13 + 2	Fetal hydrops, hygroma 9.5 mm, pleural effusion, ascites. Malposition of arms, clubfeet.	No radiographic imaging.	Genetic testing (absent movements seen with US)
30	Lethal congenital contracture syndrome 1 (GLE1)	H18 + 0	No fetal movements. Fetal edema. Contractures in upper and lower extremities. Hips flexed and knees straight. Malposition in wrists and ankles. Small chin.	No radiographic imaging.	Genetic testing (absent movements seen with US)
31	Freeman‐Sheldon syndrome (MYH3)	H20 + 5	Restricted fetal movements. Both shoulders and elbows moving, hips moving little, lower extremities not moving. Knees and ankles extended, toes flexed. Small chin.	X‐ray and CT: **Bell‐shaped chest, ribs angulated. Dislocated right hip,** hyperextended knees, **rocker‐bottom foot, dorsiflexed wrists.**	Genetic testing supported by X‐ray and CT
32	Mulibrey nanism (TRIM37)	H30 + 0	Long bones short and AC small (all ‐3SD).	No radiographic imaging.	Genetic testing
33	GRACILE (BCS1L)	H20 + 6	Long bones short (2–3 weeks smaller than GA). Echogenic and dilated bowel.	No radiographic imaging.	Genetic testing
34	GRACILE (BCS1L)	H21 + 1	Humeri and femora short and AC small. Echogenic and dilated bowel.	No radiographic imaging.	Genetic testing
35	Hydrolethalus (HYLS1)	H13 + 5	NT 3.5. Cystic changes in neck and hygroma. Abnormal brain structures (midline partly absent, Dandy‐Walker anomaly, ACC, enlarged 4. ventricle), asymmetrical face.	No radiographic imaging.	Genetic testing
36	Hydrolethalus (HYLS1)	H12 + 5	Shortened extremities. Abnormal brain structure (plexus choroidea not visible). Inverted feet and dilated renal pelvises.	X‐ray and CT. Short and **bowed femora**. Other developing long bones also abnormal.	Genetic testing

Abbreviations: AC, abdominal circumference; GA, gestational age; HC, head circumference; NT, nuchal translucency; TC, chest circumference.

Genetic diagnosis was obtained during the pregnancy in 15/36 cases (41.7%) and 14 of these were diagnosed before 24 weeks of pregnancy. Ten of these pregnancies were terminated. In 17 cases (47.2%), diagnosis was obtained after termination and in four cases (11.1%) after live birth. Overall, in less than half of the cases, genetic diagnosis was obtained during pregnancy.

### 3.2 Prenatal US Findings

**FIGURE 2:**
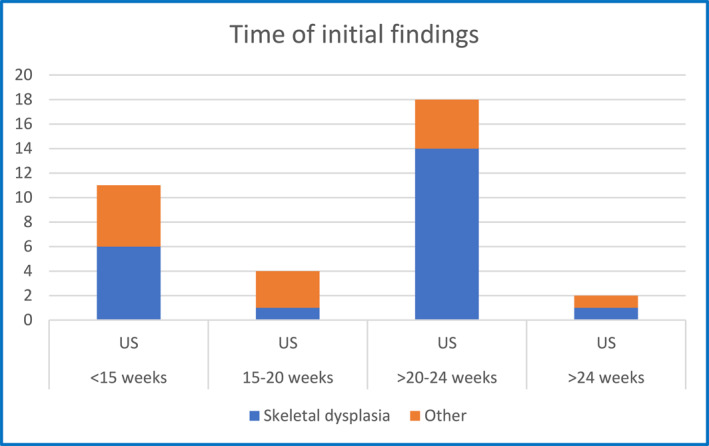
Gestational age (in weeks) when first signs of the disease were seen by US in true skeletal dysplasias and in other diseases affecting fetal growth. *N* = 35 as one case of Osteogenesis imperfecta (Sillence type 1) was not detected prenatally.

Prenatal US findings were available in all 36 cases. In 11/36 cases (30.6%), the first signs of the disease were already seen early (< 15 weeks) in pregnancy. However, almost half of these cases (5/11) had other diagnoses than skeletal dysplasias (Lethal congenital contracture syndrome [OMIM: 253310] and Hydrolethalus syndrome [OMIM: 236680]). Most of the actual skeletal dysplasias were discovered only during the second trimester (Figure 2).

US was the primary indicator of the fetal skeletal disease. It successfully identified shortening of the long bones, contractures, hydrops, other non‐skeletal anomalies, and the lack of fetal movements. Increased NT or fetal hydrops was observed in 13/36 (36.1%) cases, including two cases of skeletal dysplasia (due to *COL2A1* and *ACAN* variants) and two cases of spondylocostal dysostosis. In addition, US performed rather well in visualizing bowing of the long bones and poor ossification of the skull, and it detected some of the fractures and thoracic abnormalities. In two cases of GRACILE (Growth retardation, Aminoaciduria, Cholestasis, Iron overload, Lactic acidosis and Early Death) syndrome (OMIM: 603358), US revealed dilated and echogenic bowel along with shortening of long bones.

Based on US findings, the fetal condition was classified as lethal or severely life‐limiting in 18/36 (50%) cases. However, the methods of describing and reporting the findings tended to be variable. In five of these 18 cases, FL/AC < 0.16 was mentioned as one indicator of lethal disease. Chest circumference was not measured often; instead, chest was described to be small or bell‐shaped. In one case of Thanatophoric dysplasia, TC/AC was measured to be normal (> 0.6) but other features and FL/AC indicated lethal disease. Nine cases were described to have severe skeletal abnormalities such as very short and bowed long bones or poorly mineralized skull. The remaining cases had mainly non‐skeletal findings indicating severe disease such as fetal hydrops or lack of fetal movements. In two cases, the assessment of lethality was based solely on the molecular genetic analysis; both fetuses were diagnosed with GRACILE. All 18 cases with severe disease were discovered before 24 weeks of pregnancy. Of these 18 pregnancies, 16 were terminated. In the remaining two cases, the child was born alive but died within days after birth.

In our cohort, both lethal and non‐lethal skeletal dysplasias had normal amounts of amniotic fluid in the second trimester US and polyhydramnion was only observed in one case of non‐lethal spondyloepiphyseal dysplasia at 31 + 5 pregnancy weeks.

### 3.3 Radiographic Imaging Ex Utero

**FIGURE 3:**
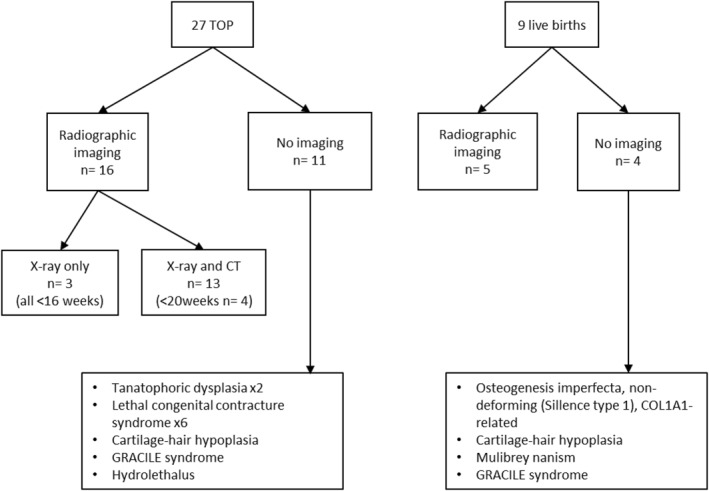
Radiographic imaging after termination of pregnancy (TOP) or after birth (during neonatal period/during first year of life).

Additional radiographic imaging (X‐ray and CT) was performed postmortem in 16 of the 27 terminated pregnancies, seven of which were first‐trimester terminations. In addition, the results of imaging studies were available from five liveborn children. Radiographic imaging was not performed during the perinatal period for children with mild phenotypes since it was unnecessary to expose them to radiation. When genetic diagnosis was confirmed and pregnancy was terminated, radiographic imaging was not routinely performed (Figure 3).

**FIGURE 4:**
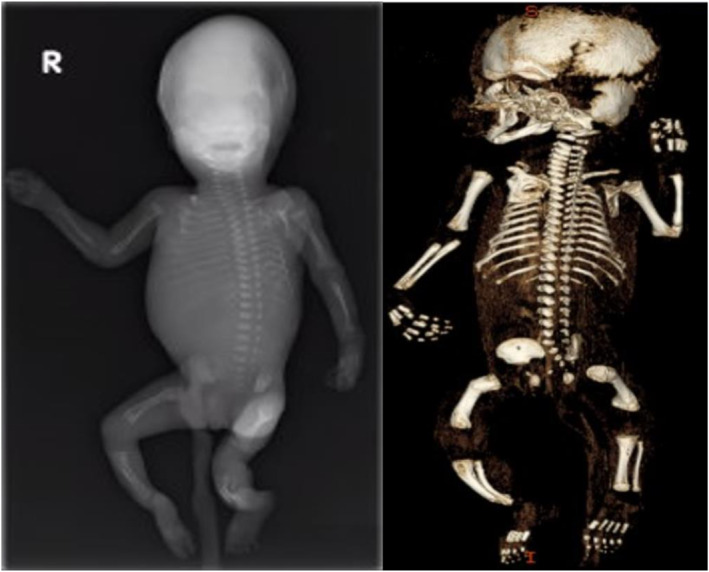
X‐ray and 3D‐CT images of a fetus (H20 + 1) with COL1A2‐related osteogenesis imperfecta after termination. Multiple rib fractures, short femora with fractures and bowed tibia can be seen.

X‐ray and especially 3D‐CT appeared to be superior to US in identifying spinal and thoracic anomalies in both the first and second trimesters of pregnancy (Table 1a and 1b). Especially, deformities, fractures and shortening of ribs were often missed with US. In nine cases, these changes had not been detected at all with US. Additionally, abnormalities and fractures of vertebrae and scapular, clavicular and acromial defects were poorly detected, and in some cases, even long bone fractures and deformities were missed with US. Occasionally bowing of long bones seen with US later appeared as fractures in radiographs, which also showed more detailed abnormalities such as widening of metaphyses and diaphyses. For instance, in one case with *COL1A2*‐related severe perinatal form of Osteogenesis imperfecta (OI) (Sillence type 2), US showed shortening and bowing of the long bones indicating skeletal dysplasia, but femoral fractures and multiple rib fractures diagnostic to severe OI were only detected with X‐ray/CT (Figure 4). In other OI cases, some fractures and for example poor ossification of the skull were visible with US, but phenotypic data provided by X‐ray/CT was much more detailed.

In the group of other diseases, radiographic imaging was not used as often because in many cases there were remarkable non‐skeletal findings leading to the diagnostic process. When imaging was performed, it revealed abnormalities that were not reported in the US. In one case of Lethal congenital contracture syndrome, X‐ray revealed a dislocated hip and in a fetus with Freeman‐Sheldon syndrome X‐ray/CT showed a bell‐shaped chest, angulated ribs, rocker‐bottom foot and dislocated right hip.

### 3.4 Disease Specific Findings

In 14/18 (77.8%) cases with true skeletal dysplasia for whom radiographic imaging was performed, X‐ray/CT was the most accurate method for detecting disease specific findings. In other diseases, genetic testing was more important as a diagnostic tool (Tables 1a and 1b).

**FIGURE 5:**
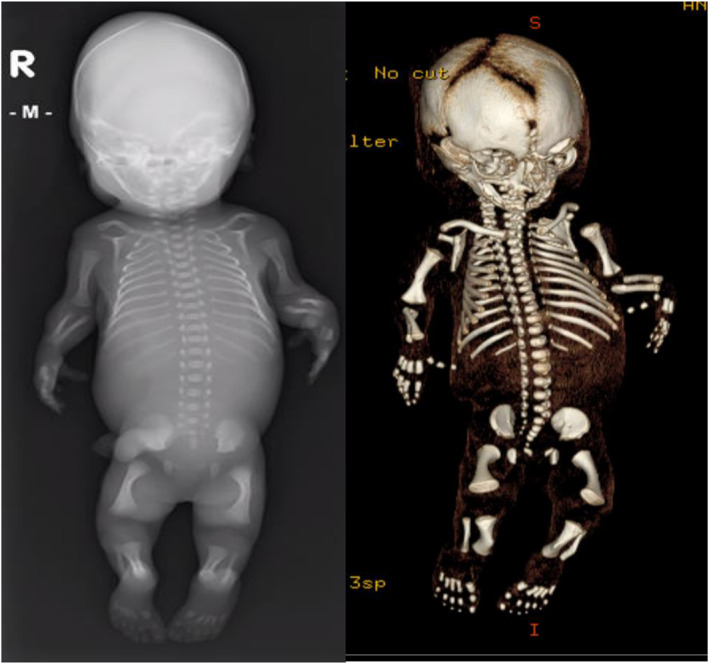
X‐ray and 3D‐CT of a terminated fetus (H20 + 6) with Diastrophic dysplasia. Long bones are short and wide and humeri and lower legs bowed. Wrists are flexed and ‘hitchhiker's thumbs' can be seen bilaterally.

All cases of Diastrophic dysplasia had the typical “hitchhiker's thumb” anomaly visible with US or post‐mortem X‐ray/CT (Figure 5). All cases with Lethal congenital contracture syndromes had increased NT and 6/7 had fetal hydrops. Surprisingly, a dilated and echogenic bowel was observed in the two fetuses with GRACILE syndrome included in this cohort.

In two cases of hydrolethalus syndrome, both fetuses had structural abnormalities of the brain but otherwise very different phenotype. The other fetus had midline abnormality with Dandy‐Walker anomaly and dilated fourth ventricle, the other one had only absent plexus choroidea. In addition, the latter fetus had shortness of long bones and bowing femora with inverted feet and dilated renal pelvises, whereas the other fetus had normal bone structure but increased NT and cystic hygroma of the neck.

## 4 Discussion

### 4.1 Post‐Mortem Radiographic Imaging

In this Finnish cohort of 36 cases of prenatally suspected fetal skeletal dysplasia, post‐mortem radiographic imaging with X‐ray and CT significantly improved diagnostic yield. The imaging was useful after both the first and second trimester terminations. These methods were superior to ultrasound especially in detecting spinal and thoracic abnormalities. X‐ray and CT also performed better in discovering fractures and deformities of long bones, thus increasing diagnostic accuracy. When imaging is performed after termination, there is no concern about radiation exposure and no need to minimize radiation dose.

Post‐mortem CT can help in evaluation of fetal skeleton especially with 3D reformats. Small targets and overlapping bones can be challenging for X‐ray only, especially after fetal demise or termination if fetus is macerated. With X‐ray, the findings can be observed only from one direction, whereas CT enables inspection from multiple directions, which can help in characterization of skeletal abnormalities. X‐ray can raise a suspicion of abnormality, but CT can confirm the finding and give more detailed information of the abnormality e.g. bowed bone might turn out as a fracture in CT. In our study, X‐ray and CT were performed parallel to enhance diagnostic quality. In line with our results, Calder and Nishimura [20, 21] have also shown the benefits of post‐mortem fetal radiographs although in their studies CT was only used occasionally.

Osteogenesis imperfecta can be challenging to diagnose with US if the fractures and deformities are not clearly visible. In our cohort, there was one case where post‐mortem X‐ray/CT revealed multiple fractures in the ribs and femora that had been missed with US. The typical findings of most severe and lethal forms of OI are usually detectable by US, but that is not the case with all prenatal‐onset types of the disease [22, 23]. X‐ray/CT can therefore improve the prenatal diagnostics of Osteogenesis imperfecta [18, 24].

### 4.2 Prenatal Ultrasound

In our cohort ultrasound performed well in visualizing shortening and bowing of the long bones, poor ossification of the skull, and some of the fractures and thoracic abnormalities. In addition, US is a very important tool in detecting non‐skeletal anomalies, fetal hydrops, contractures, and decreased fetal movements, which are important in differential diagnostics. In our cohort, most of the actual skeletal dysplasias were discovered as late as second trimester. All cases of severe or life‐limiting disease could be identified before 24 weeks of pregnancy. However, in two cases with GRACILE syndrome, US was not diagnostic, and assessment of lethality was based solely on genetic analyses. GRACILE syndrome was suspected because fetuses had significant growth restriction and this disease belongs to the Finnish disease heritage. As our study period was rather long, protocols for reporting the US findings varied and they were not always as systematic as recommended in the literature [8, 25]. However, the severity of the disease was still correctly estimated. Since the signs of the disease are seen rather late in pregnancy and phenotypes overlap, different approaches have been suggested for the earlier detection of skeletal dysplasias [26].

Polyhydramnion has been suggested as an indicator of lethal skeletal dysplasias [12, 27]. In our cohort, both lethal and non‐lethal skeletal dysplasias had normal amounts of amniotic fluid in the second trimester US. Polyhydramnion was not useful in the evaluation of lethality before 24 weeks of pregnancy.

### 4.3 Diagnostic Process

Decision‐making about the continuation of pregnancy after suspicion of fetal disease has arisen is difficult for the families. If US indicates poor prognosis or lethal disease, termination can be chosen even if the exact molecular diagnosis is unclear. This was observed in our cohort. Prolonging the situation and families' distress with additional investigations may be unnecessary if further diagnostic testing with radiographic imaging and genetic testing can be performed after termination. When the degree of severity is uncertain and exact diagnosis can help families with decision‐making or preparing for neonatal care, all available diagnostic methods should be utilized during pregnancy.

Obtaining genetic confirmation for the diagnosis of fetal skeletal dysplasia is important both for assessment of severity in an ongoing pregnancy and assessment of recurrence risk in future pregnancies. NGS‐based (Next‐generation sequencing) methods such as gene panels and whole exome sequencing have greatly improved prenatal diagnostics of skeletal dysplasias [28]. However, detailed phenotypic data are required for the interpretation of the results, especially in cases where novel or rare variants are detected. Therefore, it is recommended to use complementary imaging methods including post‐mortem radiography to obtain as much phenotypic data as possible.

### 4.4 Disease Specific Findings

Some interesting observations about rare inherited conditions affecting fetal growth were made in this study. GRACILE syndrome is known to cause considerable fetal growth retardation with small fetal abdominal diameter in second trimester [29], which was also noted in our study. Surprisingly, dilated and echogenic bowel was observed in two fetuses with GRACILE syndrome in this cohort. To our knowledge, this finding has not been reported in the literature in association with this syndrome. This finding might be related to growth restriction itself as suggested by Al‐Kouatly et al. [30], but it could also be a specific marker for this syndrome. Further studies are needed to clarify the meaning of this finding.

Lethal congenital contracture syndrome often presents during the first trimester as fetal hydrops, contractures and absent/reduced fetal movements [31]. However, in our cohort, one case was diagnosed at the second trimester screening because of short long bones.

We also identified two cases of Hydrolethalus syndrome sharing the same genotype but presenting with very different phenotypes. Both of these fetuses had structural abnormalities of the brain, which is a feature often found in this syndrome [32, 33] but the exact type of brain abnormality was different in each case and other symptoms were very distinct.

### 4.5 Prenatal CT

Many studies have shown the benefits of prenatal low‐dose or ultra‐low‐dose CT in identifying skeletal abnormalities [34, 35, 36]. In prenatal settings with lower dose of radiation, CT performed better than US in evaluating skeletal abnormalities with a reduction in radiation exposure [37, 38, 39]. However, visualization of fetal hands and feet was poor with the low dose, especially in second trimester [18, 24]. This is not the problem with post‐mortem CT when sufficient radiation dose is applicable.

Because of the fetal radiation exposure, prenatally used CT is not currently widely used nor is it used in Finland yet for this indication. With improved techniques in‐utero radiation exposure is likely to be quite small. Therefore, it has been suggested that prenatally used fetal CT should be considered when there is suspicion of severe skeletal dysplasia and diagnosis is unclear after detailed US or if molecular genetic testing is unavailable [40, 41]. Recent guidelines suggest the use of CT after 26 weeks of pregnancy [41], meaning that it is not helpful for families considering termination since the imaging is only performed after the pregnancy has already progressed past the legal time limit for termination.

### 4.6 Strengths and Limitations

Our patient cohort is very representative of the Finnish population as the study was carried out in a large tertiary hospital and all pregnant women in Finland are offered prenatal screening and possible additional testing (including genetic analyses) free of charge, resulting in high uptake of these tests. However, it has to be noted that there is considerable population‐specific variation in the genetic spectrum of fetal skeletal disease. Indeed, in this Finnish patient cohort, the diagnoses belonging to the Finnish disease heritage group covered about half of all cases with genetically confirmed diagnosis. In other populations, the percentage of diseases visible with US early in pregnancy might vary and the local protocols concerning the termination of pregnancy might also significantly affect the diagnostic process. Due to the long study period, the methods of reporting the ultrasound findings also varied, making it challenging to interpret the data.

Imaging with X‐ray and CT were performed in clinical settings over the study years without a systematic approach. Findings were reviewed concurrently, and the results were combined in the final report. Therefore, we cannot determine which proportion of the findings would have only been seen with CT. Hence, further research in this field would be beneficial.

## 5 Conclusion

Fetal radiographic imaging, especially CT, is an important method for investigating terminated or stillborn fetuses with suspected skeletal disease. As ultrasound is not able to detect all abnormalities, post‐mortem X‐ray/CT offers additional information supporting the diagnostic process. Detailed phenotypic data are important for the interpretation of the results of molecular genetic testing, especially in cases with novel or rare variants. This information is valuable in the assessment of recurrence risk in future pregnancies.

Ultrasound performs well in detecting skeletal dysplasias and severe forms of the disease. It is also an important tool in visualizing other anomalies, fetal hydrops, contractures, and lack of fetal movements, which are important in differential diagnostics as there are other diseases affecting fetal growth and mimicking skeletal disease. Despite improved imaging methods, actual skeletal dysplasias are still detected rather late, usually in second trimester of pregnancy; therefore, additional approaches and diagnostic methods are needed.

## Conflicts of Interest

The authors declare no conflicts of interest.

## Data Availability

The data that support the findings of this study are available upon reasonable request from the corresponding author.

## References

[1] I. M. Orioli , E. E. Castilla , and J. G. Barbosa‐Neto , “The Birth Prevalence Rates for the Skeletal Dysplasias,” Journal of Medical Genetics 23, no. 4 (1986): 328–332, 10.1136/jmg.23.4.328. 3746832 PMC1049699

[2] D. Krakow , “Skeletal Dysplasias,” Clinics in Perinatology 42, no. 2 (2015): 301–319, 10.1016/j.clp.2015.03.003. 26042906 PMC4456691

[3] S. Unger , C. R. Ferreira , G. R. Mortier , et al., “Nosology of Genetic Skeletal Disorders: 2023 Revision,” American Journal of Medical Genetics, Part A 191, no. 5 (2023): 1164–1209, 10.1002/ajmg.a.63132. 36779427 PMC10081954

[4] K. Rajala , E. Kasanen , S. Toiviainen‐Salo , et al., “Genetic Spectrum of Prenatally Diagnosed Skeletal Dysplasias in a Finnish Patient Cohort,” Prenatal Diagnosis 42, no. 12 (2022): 1525–1537, 10.1002/pd.6186. 35611473 PMC9796183

[5] E. Pajkrt and L. S. Chitty , “A Sonographic Approach to the Prenatal Diagnosis of Skeletal Dysplasias,” Prenatal Diagnosis 39, no. 9 (2019): 701–719, 10.1002/pd.5501. 31173381

[6] A. Khalil , E. Pajkrt , and L. S. Chitty , “Early Prenatal Diagnosis of Skeletal Anomalies,” Prenatal Diagnosis 31, no. 1 (2011): 115–124, 10.1002/pd.2676. 21210484

[7] G. Gaffney , N. Manning , P. A. Boyd , V. Rai , S. Gould , and P. Chamberlain , “Prenatal Sonographic Diagnosis of Skeletal Dysplasias—A Report of the Diagnostic and Prognostic Accuracy in 35 Cases,” Prenatal Diagnosis 18, no. 4 (1998): 357–362, 10.1002/(sici)1097-0223(199804)18:4<357::aid-pd276>3.0.co;2-0. 9602482

[8] A. E. Noel and R. N. Brown , “Advances in Evaluating the Fetal Skeleton,” International Journal of Women's Health 6 (2014): 489–500, 10.2147/ijwh.s47073. PMC4027851 24868173

[9] D. Krakow , Y. Alanay , L. P. Rimoin , et al., “Evaluation of Prenatal‐Onset Osteochondrodysplasias by Ultrasonography: A Retrospective and Prospective Analysis,” American Journal of Medical Genetics, Part A 146A, no. 15 (2008): 1917–1924, 10.1002/ajmg.a.32269. 18627037 PMC2713784

[10] J. S. Dashe , D. D. McIntire , and D. M. Twickler , “Effect of Maternal Obesity on the Ultrasound Detection of Anomalous Fetuses,” Obstetrics & Gynecology 113, no. 5 (2009): 1001–1007, 10.1097/aog.0b013e3181a1d2f5. 19384114

[11] T. Schramm , K. P. Gloning , S. Minderer , et al., “Prenatal Sonographic Diagnosis of Skeletal Dysplasias,” Ultrasound in Obstetrics and Gynecology 34, no. 2 (2009): 160–170, 10.1002/uog.6359. 19548204

[12] K. S. Milks , L. M. Hill , and K. Hosseinzadeh , “Evaluating Skeletal Dysplasias on Prenatal Ultrasound: An Emphasis on Predicting Lethality,” Pediatric Radiology 47, no. 2 (2017): 134–145, 10.1007/s00247-016-3725-5. 27904917

[13] A. Stembalska , L. Dudarewicz , and R. Smigiel , “Lethal and Life‐Limiting Skeletal Dysplasias: Selected Prenatal Issues,” Advances in Clinical and Experimental Medicine 30, no. 6 (2021): 641–647, 10.17219/acem/134166. 34019743

[14] N. Chandler , S. Best , J. Hayward , et al., “Rapid Prenatal Diagnosis Using Targeted Exome Sequencing: A Cohort Study to Assess Feasibility and Potential Impact on Prenatal Counseling and Pregnancy Management,” Genetics in Medicine 20, no. 11 (2018): 1430–1437, 10.1038/gim.2018.30. 29595812

[15] A. C. Jelin , K. Blakemore , S. Trebes , et al., “Molecular Testing Strategies in the Evaluation of Fetal Skeletal Dysplasia,” Journal of Maternal‐Fetal and Neonatal Medicine 35, no. 14 (2022): 2788–2794, 10.1080/14767058.2020.1802715. 32752906 PMC7858696

[16] L. A. Gilligan , M. A. Calvo‐Garcia , K. N. Weaver , and B. M. Kline‐Fath , “Fetal Magnetic Resonance Imaging of Skeletal Dysplasias,” Pediatric Radiology 50, no. 2 (2020): 224–233, 10.1007/s00247-019-04537-8. 31776601

[17] R. U. Bisht , M. V. Belthur , I. M. Singleton , and L. F. Goncalves , “Accuracy of Multimodality Fetal Imaging (US, MRI, and CT) for Congenital Musculoskeletal Anomalies,” Children 10, no. 6 (2023): 1015, 10.3390/children10061015. 37371247 PMC10297094

[18] T. Victoria , M. Epelman , M. Bebbington , et al., “Low‐dose Fetal CT for Evaluation of Severe Congenital Skeletal Anomalies: Preliminary Experience,” Pediatric Radiology 42, no. Suppl 1 (2012): S142–S149, 10.1007/s00247-011-2175-3. 22395726

[19] E. Barkova , U. Mohan , D. Chitayat , et al., “Fetal Skeletal Dysplasias in a Tertiary Care Center: Radiology, Pathology, and Molecular Analysis of 112 Cases,” Clinical Genetics 87, no. 4 (2015): 330–337, 10.1111/cge.12434. 24863959

[20] A. D. Calder and A. C. Offiah , “Foetal Radiography for Suspected Skeletal Dysplasia: Technique, Normal Appearances, Diagnostic Approach,” Pediatric Radiology 45, no. 4 (2015): 536–548, 10.1007/s00247-014-3130-x. 25173408

[21] G. Nishimura , A. Handa , O. Miyazaki , et al., “Prenatal Diagnosis of Bone Dysplasias,” British Journal of Radiology 96, no. 1147 (2023): 20221025, 10.1259/bjr.20221025. 37351952 PMC10321247

[22] B. V. Parilla , E. A. Leeth , M. P. Kambich , P. Chilis , and S. N. MacGregor , “Antenatal Detection of Skeletal Dysplasias,” Journal of Ultrasound in Medicine 22, no. 3 (2003): 255–258, 10.7863/jum.2003.22.3.255. 12636325

[23] J. S. Weaver , J. W. Revels , J. M. Elifritz , B. Whitlow , M. Retrouvey , and S. S. Wang , “Clinical Manifestations and Medical Imaging of Osteogenesis Imperfecta: Fetal through Adulthood,” Acta Medica Academica 50, no. 2 (2021): 277–291, 10.5644/ama2006-124.343. 34847680

[24] T. Victoria , M. Epelman , B. G. Coleman , et al., “Low‐dose Fetal CT in the Prenatal Evaluation of Skeletal Dysplasias and Other Severe Skeletal Abnormalities,” American Journal of Roentgenology 200, no. 5 (2013): 989–1000, 10.2214/ajr.12.9722. 23617480

[25] T. Victoria , X. Zhu , R. Lachman , et al., “What Is New in Prenatal Skeletal Dysplasias?,” American Journal of Roentgenology 210, no. 5 (2018): 1022–1033, 10.2214/ajr.17.19337. 29528710

[26] Y. Li , H. Zhou , X. Yang , D. Li , and L. Can , “The Application of Crown‐Chin Length to Crown‐Rump Length Ratio in Predicting Fetal Skeletal Dysplasia at First Trimester,” Journal of Ultrasound in Medicine 41, no. 10 (2022): 2497–2504, 10.1002/jum.15936. 34978346

[27] D. B. Nelson , J. S. Dashe , D. D. McIntire , and D. M. Twickler , “Fetal Skeletal Dysplasias: Sonographic Indices Associated With Adverse Outcomes,” Journal of Ultrasound in Medicine 33, no. 6 (2014): 1085–1090, 10.7863/ultra.33.6.1085. 24866616

[28] Y. Liu , L. Wang , Y. K. Yang , et al., “Prenatal Diagnosis of Fetal Skeletal Dysplasia Using Targeted Next‐Generation Sequencing: An Analysis of 30 Cases,” Diagnostic Pathology 14, no. 1 (2019): 76, 10.1186/s13000-019-0853-x. 31299979 PMC6626426

[29] V. Fellman , I. Visapaa , M. Vujic , U. B. Wennerholm , and L. Peltonen , “Antenatal Diagnosis of Hereditary Fetal Growth Retardation With Aminoaciduria, Cholestasis, Iron Overload, and Lactic Acidosis in the Newborn Infant,” Acta Obstetricia et Gynecologica Scandinavica 81, no. 5 (2002): 398–402, 10.1034/j.1600-0412.2001.810504.x. 12027811

[30] H. B. Al‐Kouatly , S. T. Chasen , A. K. Karam , R. Ahner , and F. A. Chervenak , “Factors Associated With Fetal Demise in Fetal Echogenic Bowel,” American Journal of Obstetrics and Gynecology 185, no. 5 (2001): 1039–1043, 10.1067/mob.2001.117641. 11717629

[31] K. Vuopala and R. Herva , “Lethal Congenital Contracture Syndrome: Further Delineation and Genetic Aspects,” Journal of Medical Genetics 31, no. 7 (1994): 521–527, 10.1136/jmg.31.7.521. 7966188 PMC1049973

[32] P. Ammala and R. Salonen , “First‐Trimester Diagnosis of Hydrolethalus Syndrome,” Ultrasound in Obstetrics and Gynecology 5, no. 1 (1995): 60–62, 10.1046/j.1469-0705.1995.05010060.x. 7850595

[33] T. J. de Ravel , M. C. van der Griendt , P. Evan , and C. A. Wright , “Hydrolethalus Syndrome in a Non‐Finnish Family: Confirmation of the Entity and Early Prenatal Diagnosis,” Prenatal Diagnosis 19, no. 3 (1999): 279–281, 10.1002/(sici)1097-0223(199903)19:3<279::aid-pd518>3.0.co;2-l. 10210131

[34] R. Ruano , M. Molho , J. Roume , and Y. Ville , “Prenatal Diagnosis of Fetal Skeletal Dysplasias by Combining Two‐Dimensional and Three‐Dimensional Ultrasound and Intrauterine Three‐Dimensional Helical Computer Tomography,” Ultrasound in Obstetrics and Gynecology 24, no. 2 (2004): 134–140, 10.1002/uog.1113. 15287049

[35] M. Cassart , A. Massez , T. Cos , et al., “Contribution of Three‐Dimensional Computed Tomography in the Assessment of Fetal Skeletal Dysplasia,” Ultrasound in Obstetrics and Gynecology 29, no. 5 (2007): 537–543, 10.1002/uog.4001. 17444568

[36] G. Mace , P. Sonigo , V. Cormier‐Daire , et al., “Three‐dimensional Helical Computed Tomography in Prenatal Diagnosis of Fetal Skeletal Dysplasia,” Ultrasound in Obstetrics and Gynecology 42, no. 2 (2013): 161–168, 10.1002/uog.12298. 22945478

[37] O. Miyazaki , G. Nishimura , H. Sago , T. Horiuchi , S. Hayashi , and R. Kosaki , “Prenatal Diagnosis of Fetal Skeletal Dysplasia with 3D CT,” Pediatric Radiology 42, no. 7 (2012): 842–852, 10.1007/s00247-012-2381-7. 22532233

[38] M. Waratani , F. Ito , Y. Tanaka , A. Mabuchi , T. Mori , and J. Kitawaki , “Prenatal Diagnosis of Fetal Skeletal Dysplasia Using 3‐dimensional Computed Tomography: A Prospective Study,” BMC Musculoskeletal Disorders 21, no. 1 (2020): 662, 10.1186/s12891-020-03663-x. 33032557 PMC7545947

[39] R. Imai , O. Miyazaki , T. Horiuchi , et al., “Ultra‐Low‐Dose Fetal CT With Model‐Based Iterative Reconstruction: A Prospective Pilot Study,” American Journal of Roentgenology 208, no. 6 (2017): 1365–1372, 10.2214/ajr.16.17593. 28463542

[40] E. J. Snyder , J. S. Moldenhauer , and T. Victoria , “Prenatal Diagnosis of Skeletal Dysplasias: What Can CT Do for You?,” Fetal Diagnosis and Therapy 50, no. 2 (2023): 61–69, 10.1159/000528692. 36948169

[41] P. Bach , M. Cassart , M. Chami , C. Garel , and M. Panuel , “Exploration of the Fetal Skeleton by Ultra‐Low‐Dose Computed Tomography: Guidelines From the Fetal Imaging Task Force of the European Society of Paediatric Radiology,” Pediatric Radiology 53, no. 4 (2023): 621–631, 10.1007/s00247-022-05487-4. 36028720

